# Impact of Guided Implant Dentistry on Patient Quality of Life, Satisfaction, and Psychological Well-Being: A Systematic Review

**DOI:** 10.3390/jcm14186638

**Published:** 2025-09-20

**Authors:** Daniela García-Valdez, Eugenio Velasco-Ortega, Iván Ortiz-Garcia, Loreto Monsalve-Guil, José López-López, Enrique Núñez-Márquez, Nuno Matos-Garrido, Álvaro Jiménez-Guerra, Jesús Moreno-Muñoz, José Luis Rondón-Romero

**Affiliations:** 1 Comprehensive Dentistry for Adults and Gerodontology, Faculty of Dentistry, University of Seville, 41009 Seville, Spain; dangarval@alum.us.es (D.G.-V.); evelasco@us.es (E.V.-O.); lomonsalve@hotmail.es (L.M.-G.); enrique_aracena@hotmail.com (E.N.-M.); nunogarrido@orallagos.pt (N.M.-G.); alopajanosas@hotmail.com (Á.J.-G.); je5us@hotmail.com (J.M.-M.); jolurr001@hotmail.com (J.L.R.-R.); 2 Service of the Medical-Surgical Area of Dentistry Hospital, Faculty of Dentistry, University of Barcelona, 08007 Barcelona, Spain

**Keywords:** diagnostic, guided implantology, patient perception, satisfaction, quality of life, computer-assisted implant surgery, dynamic navigation

## Abstract

**Introduction:** Oral implantology, a modern approach to rehabilitating edentulous patients, has advanced significantly with digital technologies, notably computer-guided surgery. This technique is considered precise and predictable. However, it is essential to assess this technique from the patient’s perspective, focusing on its impact on quality of life and satisfaction. **Methods:** A literature search was conducted in PubMed, Embase, and CINAHL up to January 2025. Clinical trials and case series studies were included. Studies conducted on partially or fully edentulous patients were selected for inclusion. The studies included static or dynamic guided oral implant treatments, as well as conventional treatments, and evaluated patient-reported outcomes, specifically perceived satisfaction and quality of life. A qualitative synthesis of the findings was performed, and the quality of the included studies was assessed using the Newcastle–Ottawa Scale (NOS). **Results:** A total of twelve studies were included. The most commonly used questionnaires for evaluation were the Visual Analog Scale (VAS), Oral Health-Related Quality of Life (OHQoL), and Oral Health Impact Profile (OHIP). Computer-guided implantology appears to be a valid and predictable technique for dental implant placement. It is associated with a reduced intraoperative and postoperative pain. Some studies, however, did not identify significant differences compared with conventional implant surgery. **Conclusions:** Guided oral implantology is a viable option for oral rehabilitation in edentulous patients, offering benefits in surgical precision, pain reduction, and patient experience. Its effects on surgical time and overall patient satisfaction, however, warrant further investigation.

## 1. Introduction

Healthcare has undergone a paradigm shift towards a patient-centered approach [1]. This shift has led to a broad consensus, across researchers and clinical dental practice, on integrating assessments of oral health status from the patient’s perspective [2]. This approach not only deepens understanding of patients’ needs and concerns but also supports the development of more effective and personalized strategies for dental care. Patient-reported outcomes primarily assess the of oral health on daily life and oral health-related quality of life (OHRQoL), and they also measure patient satisfaction with oral health status, offering a more comprehensive view of overall well-being [3].

Edentulism profoundly impairs patient’s quality of life. It affects millions worldwide and is classified as a chronic disease that poses a major challenge for dental rehabilitation (International Classification of Functioning, Disability and Health (ICF) [4].

Approximately 50 years ago, osseointegrated implants were introduced as a treatment option for edentulous patients who experienced difficulties with conventional complete dentures. Such prostheses were associated with higher rates of alveolar bone resorption and biomechanical problems, including poor retention and stability, that can compromise masticatory efficiency. Moreover, their limited aesthetic properties rendered them less psychologically acceptable to patients [5,6].

In this context, the introduction of dental implants marked a milestone in the evolution of oral rehabilitation in clinical practice. Today, implant placement is among the most reliable and predictable treatment options for edentulism, offering an alternative that not only improves bone and masticatory function but also addresses patient needs for comfort, aesthetics, phonetics, stability, and prosthesis retention [7,8].

Over the past decade, the digital revolution, driven by continuous technological advancement and innovation, has profoundly transformed dental practice through the integration of 3D technologies for diagnosis and treatment planning. These include intraoral scanners, facial scanners, cone-beam computed tomography (CBCT), 3D printers, and CAD/CAM (computer-aided design/computer-assisted manufacturing) milling machines.

Enabled by these technologies, computer-guided surgery emerged in oral implantology as an innovative approach that has reshaped contemporary practice. It has driven a shift from conventional procedures to highly specialized techniques that enhance the precision and safety of implant placement, resulting in significantly higher survival rates. Virtual planning focuses not only on optimizing implant positioning but also on meeting prosthetic requirements and considerations [9].

These aforementioned techniques include static computer-assisted implant surgery (s-CAIS) and dynamic computer-assisted implant surgery, also known as dynamic navigation (d-CAIS) which can sometimes include robotic computer-aided implant system (r-CAIS) [10,11,12]. Static systems use laboratory-fabricated surgical guides, produced by milling or CAD/CAM-based stereolithography. By contrast, dynamic systems provide real-time navigation that visualizes the drill’s position relative to patient anatomy, enabling controlled, guided drilling in accordance with the preoperative surgical plan [13].

Given the growing interest in computer-guided implantology, and despite numerous studies addressing its clinical success, it is essential to systematically evaluate how these techniques influence patient perception, including quality of life, satisfaction, and psychological well-being, all of which contribute to the overall outcome of the treatment [14].

This study aims to analyze the existing literature on patient-reported outcomes of computer-guided oral implantology treatments, including quality of life and treatment satisfaction.

## 2. Materials and Methods

A systematic literature review was conducted in accordance with PRISMA 2020 (Preferred Reporting Items for Systematic Reviews and Meta-Analyses) standards [14,15].

On 30 May 2025, a literature search was carried out in the biomedical databases PubMed (Medline), Embase, and CINAHL. The search combined keywords and MeSH terms: “guided dental implants,” “computer-assisted dental surgery,” “oral implantology,” “patient perception,” “psychological implications,” and “quality of life” using the Boolean operators “OR” and “AND.” In addition, reference lists of the included studies were manually reviewed.

Articles published in peer-reviewed journals were eligible if they met the following PECOS criteria: Population, Exposure, Comparison, Outcome, Study Design.

(a) Population: studies conducted on adults (18 years and older) who were partially or completely edentulous; (b) Exposure: studies including patients who received static or dynamic computer-guided oral implantology treatment; (c) Comparison: studies that included patients who received conventional implant treatments and/or specific techniques of computer-guided surgery: static computer-assisted implant surgery (s-CAIS) and dynamic navigation (d-CAIS) (d) Outcome: patient-perception measures, specifically satisfaction and quality of life, including variables such as esthetics, comfort, masticatory function, phonetics, surgical time, oral hygiene, and intra- and postoperative pain; (e) Study Design: randomized clinical trials and case series studies.

Studies were excluded if they lacked a comparison group using computer-guided implantology techniques, included populations receiving prior psychological/psychiatric treatments that could influence results, or evaluated only guided implantology in overdentures. Systematic and narrative reviews were excluded. Conference abstracts, editorials, commentaries, and in vitro or cadaveric studies were also not considered.

### 2.1. Selection and Data Extraction

Study selection and data extraction were performed independently by two researchers. After removing duplicates, titles and abstracts were screened against the inclusion and exclusion criteria; potentially eligible records underwent full-text review. Any discrepancies were resolved through discussion, with adjudication by a third reviewer when necessary.

Once the final set of studies was identified, two authors independently extracted data using a prespecified, piloted extraction form adapted from the STROBE checklist [16]. A third author verified the extracted data.

The first table summarizes bibliographic and study characteristics (author, year of publication, country, sample size, number of implants placed), study design, patient characteristics (age and sex), main conclusions, and conflict-of-interest statements.

The second table records clinical treatment variable: type of guided surgery, comparison group, flap versus flapless approach, and prosthetic loading time.

The third table captures patient-perception outcomes. It also includes the questionnaire used for evaluation, aspects and variables assessed, and the timing of the measurement.

### 2.2. Data Analysis

For clinical evaluation studies, a descriptive synthesis of the extracted results was performed as well as a quantitative analysis when possible.

### 2.3. Quality of Included Studies

Two scales were used to assess methodological quality, depending on study type. For clinical trials, two independent reviewers applied the Jadad scale [17], which evaluates randomization, double-blinding, and accounting for withdrawals or dropouts, with a maximum score of 5 points. Quality thresholds were defined as 0–3 (low quality) and 4–5 (high quality). For case series, we used the Joanna Briggs Institute 2017 critical appraisal tool for case series, which assesses variables such as clearly defined inclusion criteria, case selection, follow-up duration, confounding factor management, and statistical methods. Studies were graded as >70% (good), 50–70% (moderate), and <50% (low methodological quality) [18].

## 3. Results

### 3.1. Search Results

**Figure 1:**
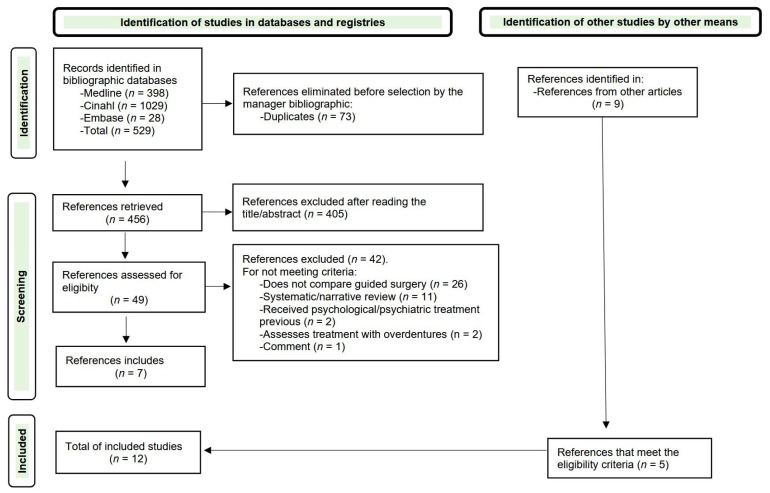
Study selection flowchart.

The search identified 515 studies. After removal of duplicates, 442 studies remained for title and abstract screening. Of these, 49 underwent full-text review; 7 met the eligibility criteria and were included. Screening of reference list yielded an additional nine potential studies, five of which were included. The flow diagram is shown in Figure 1, and the detailed search strategies are provided in Table 1.

### 3.2. Characteristics of the Included Studies

Of the twelve studies included in the systematic review, nine were clinical trials (one nonrandomized [19]) and three were case series [20,21,22].

The studies were conducted between 2006 and 2023. Study populations were drawn from a wide range of countries, including Belgium [23,24], Spain [19,25], Italy [20,26], Switzerland [27], India [9], Brazil [28], France [21], Thailand [29], and The Netherlands [22]. Outcome assessment periods from seven days to a maximum of one year. Follow-up focused exclusively on patient-reported variables related to quality of life and satisfaction in relation to the surgical procedure, both intraoperatively and in the immediate postoperative period. These data are summarized in Table 2.

None of the twelve studies reported conflicts of interest. Regarding funding, six studies declared having received financial support [19,20,23,24,27,28]: five reported private funding [20,23,24,27,28] and one of these also reported public funding [27]. Three studies stated they had no funding [9,25,26], and three did not report funding information [21,22,29].

### 3.3. Sample Size and Sociodemographic Characteristics

All studies included both male and female patients, except three in which sex was not reported [9,22,27]. All patients were at least 18 years old, and mean ages ranged from 48 to 82 years.

Sample size ranged from 10 [28] to 104 patients [19]. The number of implants placed ranged from 20 [28] to 399 [19]. Across all studies, the combined sample comprised 622 patients and 1929 implants.

### 3.4. Quality Assessment

The methodological quality of the included studies was as follows: among clinical trials evaluated with the Jadad scale, scores ranged from 1 to 5, with a mean of 2.77, indicating generally low quality. Among case series assessed with the Joanna Briggs Institute scale, scores ranged from 66% to 89%, with a mean of 77.7%. This indicates the high-quality of execution for the included studies. This information is provided in detail in Table 2. Regarding the RCTs, only the work of Frizzera et al., 2011 [27] had a score of 5, but it only analyses visual scales and included a small sample, 10 patients and 20 implants.

### 3.5. Results of the Clinical Variables from the Included Studies

Based on the type of computer-guided surgery performed, nine studies used static computer-assisted implant surgery (s-CAIS) [19,20,21,22,23,24,26,27,28], two used dynamic navigation (d-CAIS) [9,25], and one included both approaches [29].

Comparison groups varied by surgical technique (conventional, static [s-CAIS], or dynamic [d-CAIS]) and by specific clinical variables analyzed. In five studies, implant placement with s-CAIS was compared with conventional freehand surgery [21,23,24,26,28]. In the study that included both guided surgery techniques (s-CAIS and d-CAIS), the comparison group underwent conventional freehand surgery [29].

In two studies, d-CAIS was compared with conventional freehand surgery [9,25]. One study did not include a comparison group and evaluated only s-CAIS. One study did not include a comparison group and used only static guided surgery (s-CAIS) as the treatment technique for implant placement [20].

In the remaining studies, the comparison groups included both guided and conventional techniques but were subdivided based on specific variables, such as the use of radiographic and surgical stents, flap versus flapless surgery, and the type of prosthetic loading applied [19,22,27].

Another important clinical variable considered in the studies was whether a surgical flap was performed. In most cases, flap surgery was performed with the conventional technique [9,19,21,27,28]. In other cases, a flap was created due to various reasons, such as lack of mucosal support, or in patients requiring prior bone regeneration regardless of the surgical technique used [20,23,24,29], or when the study was specifically evaluating outcomes based on flap versus flapless procedures [22].

The most commonly used software programs in the included studies for computer-guided implant placement were Noble Biocare [20,22,26], Dentsply Systems [23,24,27], and Navident [9,25]. This information is summarized in Table 3.

### 3.6. Results of the Instruments Used to Assess Patient Perception

Across the twelve included studies, patient perceptions of the surgical intervention were assessed using the following instruments: Oral Health Impact Profile (OHIP) (*n* = 3) [22,24,25], Visual Analog Scale (VAS) for pain (*n* = 7) [9,21,23,25,26,27,28,29], Visual Analog Scale for quality of life (*n* = 3) (19, 20, 27), Health-Related Quality of Life instrument (HRQoL) (*n* = 2) [19,24], McGill Pain Questionnaire (MPQ-DLV) (*n* = 1) [24], adapted Likert scale (*n* = 1) [29], Patient-Related Experience Measures (PREMs) (*n* = 1) [9], Impact of Event Scale-Revised (IES-R) (*n* = 1) [22], Dental Anxiety Inventory (s-DAI) (*n* = 1) [22], Ordinal Pain Scale (*n* = 1) [26], and study-specific questionnaires (*n* = 2) [20,26]. Details are provided in Table 4

### 3.7. Results of the Validation of Patient Perception

Patient-perception outcomes related to surgical approach to implant placement were evaluated using the previously described questionnaires and scales [30,31,32,33,34,35,36,37]. In several studies, investigators also employed study-specific questionnaires to assess the outcomes [20,21,22,23,24,25,26].

The questionnaires assessed symptoms and intraoperative/postoperative comfort [9,21,23,25,26,27,28,29], patient satisfaction [9,19,20,22,24,25,26,28,29], and treatment-related perceived quality of life [19,22,23,24,25]. Other factors assessed were perception of the duration of the surgery [27], expectations [9], and preoperative and intraoperative anxiety [22].

The most commonly used outcome measures were the Visual Analog Scale (VAS) [32], both for intraoperative and postoperative pain and for patient satisfaction, the Oral Health Impact Profile (OHIP) [30], and the Oral Health-Related Quality of Life (HRQoL) questionnaire [31].

Most questionnaires evaluated similar variables. In some studies, these were presented more broadly as domains (functional limitation, physical pain, psychological discomfort, physical disability, social disability, and other causes of discomfort) [24], whereas other studies assessed more specific variables, including swallowing, phonetics, oral hygiene, sleeping, smiling, work-related functions, anxiety, esthetics, and cost [19,20,22,26].

The timing of questionnaire administration and follow-up varied widely across the included studies. Questionnaires were administered before surgery [9,19,24,25,28]; immediately after the procedure [25,27]; during the postoperative period, ranging from 3 to 15 days after surgery [9,21,22,23,25,26,28,29]; or with follow-up periods of six months to five years post-surgery (19,24,26). In nearly all studies, except one [27], questionnaires were administered more than once at different time points. This information is shown in Table 5.

### 3.8. Results of the Study Conclusions

Key findings from the included studies are summarized below. Regarding satisfaction, two studies reported that computer-guided implantology and flapless approaches were associated with greater patient satisfaction and improved quality of life [19,28], as well as a high level of accuracy in implant placement [25]. However, two studies found no significant differences between guided techniques and conventional freehand methods [25,29].

With respect to pain, four studies observed that computer-guided and/or flapless implant placement was associated with lower intensity and shorter duration of postoperative pain and inflammation than conventional freehand techniques [9,21,23,28], which may reduce medication consumption [28]. By contrast, two studies found no significant differences in pain or inflammation between conventional and guided techniques for implant placement [25,29]. Regarding flap creation in guided surgeries, one study observed that no postoperative pain was reported [22].

**Figure 2:**
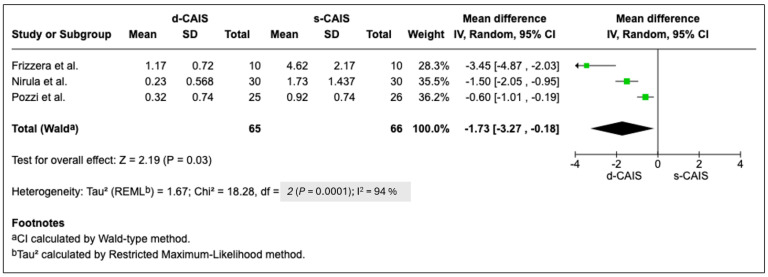
Forest plot.

Quantitative analysis was possible in only three studies, and solely with respect to pain outcomes: Pozzi et al. [25], Frizzera et al. [27], and Nirula et al. [28], yielding *p* = 0.03 (Figure 2). In the remaining studies, quantitative analysis was not feasible, either because a personalized questionnaire was used [29], data were presented only in graphical form [24,26], specific variables were analyzed [18], or different groups and measurement scales were employed [22].

Three studies concluded that treatments for implant placement using computer-guided surgery techniques are valid and predictable methods, as well as technologies with high patient preference and acceptance [9,26,27].

Finally, another variable considered was treatment planning, which was performed using CBCT and dedicated implant planning software. In one study, this technique proved to be very useful, especially for planning complex cases [20]. However, another study found no significant differences in the implementation of these software tools [26]. Despite technological advances, factors such as operator experience [20] and the time invested in these procedures can affect the efficiency of the techniques employed [27]. These results are detailed in Table 6.

## 4. Discussion

The findings of this review highlight the generally positive impact of guided oral implantology on patients’ perceptions of quality of life, satisfaction, and psychological well-being. However, results are not uniform across studies. Merli et al. [19] and Nirula et al. [28] reported improvements in patient satisfaction and quality of life, along with reductions in postoperative pain, particularly with dynamic techniques. Pozzi et al. [25] only took into account the difference in pain reduction, but not in the other parameters analyzed. Andngkawong et al. [29] and Jorba-García et al. [24] found no differences across the outcomes analyzed. Similarly, Yeo et al. [38] concluded that there is no evidence that CAIS improves patient engagement or confidence, as no intra- or postoperative differences were found. Moreover, even recent research focused on the accuracy of the three CAIS options (static, s-CAIS, dynamic, d-CAIS, and robotic r-CAIS) did not evaluate patient satisfaction [39]. By contrast, a reduction in clinician stress appears to be a consistent, as analyzed by Ashy LM [40], although implementation might be limited by the cost of the equipment [41]. This finding is further supported in favor of d-CAIS, as quantitative analysis was possible in at least three studies [25,27,28], showing a significant p value in the evaluation of pain outcomes.

Across the reviewed literature, the most frequently used questionnaires to assess these parameters were the Visual Analogue Scale (VAS), Oral Health-Related Quality of Life (OHQoL), and the Oral Health Impact Profile (OHIP). Overall, the use of digital technologies, such as computer-guided implant surgery in its static (s-CAIS) and dynamic (d-CAIS) forms, is indicated to optimize the planning and placement of dental implants [38,39], although it is rarely clearly analyzed how much the patient experience improves. One benefit that is more consistently reported and aligns with our findings in this review is the reduction in pain, with correspondingly lower medication consumption [18,20,21]. Nevertheless, Vercruyssen et al. [23], with a year of follow-up and using the OHIP-49, did not find differences. Jorba-García et al. [24] reported the same but using the OHIP-14 and the VAS.

Several studies found no significant differences relative to conventional implant surgery, underscoring the need to consider additional variables such as the operator’s experience and individual patient expectations [19,27]. Patients’ perceptions of shorter operative and recovery times with guided implantology should also be considered [28]. Notably, some studies emphasized that surgical duration may increase with case complexity and operator experience, potentially affecting overall satisfaction [20,25]. This aligns with prior studies indicating that procedure length is a key determinant of patient experience, in part by reducing anxiety and fear associated with invasive procedures [42,43,44]. Many of these issues were addressed in the review by Yeo et al. [38], which concluded that quantitative outcomes show no differences and that the qualitative literature on patient satisfaction lacks sufficiently well-designed studies to support firm conclusions.

Similarly, several studies reported no significant differences in patient-reported satisfaction or pain between using guided techniques and conventional surgery, while pointing out limitations when using guided techniques. This suggests that heterogeneity in clinical management and subjective patient factors may influence outcomes. Consistent with Aghaloo et al. [45], guided surgery can reduce operative time and postoperative inflammation, yet not all patients perceive meaningful improvements in long-term comfort compared to traditional techniques. This variability could be explained by individual differences in pain perception and implant adaptation [45]. Although CAIS systems demonstrably improve placement accuracy [12,18,46,47], adoption must also weight economic cost and the potential for sponsorship bias, given that many studies are industry [46,47].

An important aspect to consider is that dental patient-reported outcome measures (dPROMs) are widely used in various areas of oral health and well-being analysis. In fact, some of the most extensively studied are the OHQoL and the OHIP [48,49,50,51]. It is always essential to apply them using sound methodology in order to achieve the most reliable results possible [52]. However, in general terms, their limitations include incomplete knowledge regarding their dimensionality, which affects their validity, and the lack of a specified recall period, which reduces their clinical applicability [a]. One way to address these limitations is to assess the four dimensions jointly: Oral Function, Orofacial Pain, Orofacial Appearance, and Psychosocial Impact [48,53].

In our review, HRQoL was used by two authors [18,22], and although the studies were not comparable, the results were contradictory. Regarding OHIP, it was analyzed in three studies [21,23,24]. The study by Lindeboom & Van Wijk [21], a case series, reported improvement for d-CAIS, while the others showed disparate outcomes, with special mention of the study by Jorba-García et al. [24], which applied both OHIP and VAS. Nevertheless, the heterogeneity with other studies that used the VAS system prevented us from conducting a quantitative analysis.

Focusing on the VAS method, in our review, it was the most frequently used system: in eight studies for the assessment of pain [20,22,24,25,26,27,28,29] and in three studies for quality of life [18,26,27]. As mentioned previously, we were able to conduct a quantitative analysis in three of them (Figure 2) [25,27,28]. Although methodologically sensitive, the use of dPROMs has been valuable in numerous studies on oral implantology [54,55,56]. We highlight the work of Abou-Ayashe et al. [57], which presents an interesting meta-analysis of 28 studies reporting dPROs from 1457 edentulous patients, analyzing both OHIP and VAS. The authors noted that both tests, in studies where they were applied together, performed similarly. However, they also pointed out in their discussion that it is common for summary scores of validated multi-item questionnaires (e.g., OHIP) to be considered equivalent to the results of individual questions (e.g., single VAS items). This practice is, at the very least, questionable, as it may introduce bias due to the variable psychometric quality of dPROMs and the diversity of dPRO concepts [57].

This study presents several strengths. First, it synthesizes recent scientific literature, situating the findings within the current state of the art in guided implantology. Second, it highlights the benefits of integrating digital technologies into implantology, offering a modern, clinically relevant, and evidence-based perspective for clinical practice. Third, its emphasis on patient-reported outcomes provides a patient-centered approach, focused on well-being and quality of life, which are fundamental aspects of contemporary dentistry. Finally, most included studies are randomized clinical trials, providing a high level of evidence.

However, this study also has important limitations. Most trials received low Jadad scale scores, indicating methodological shortcomings, particularly in data blinding. Additionally, substantial heterogeneity in study design, outcomes, and statistical analysis hampers comparability and complicates interpretation. Several studies had small sample sizes and short, highly variable follow-up durations, limiting the generalizability of the findings. It is also important to note that most studies lacked double blinding, which could introduce bias among participants and potentially generate responses based on previous experiences or anxiety levels. Collectively, these issues precluded a meaningful meta-analysis. It is also noteworthy that three of the included studies were case series, which further weakens the strength of the evidence. Moreover, many studies were industry funded, introducing a potential risk of sponsorship bias.

Finally, the lack of longitudinal research evaluating the long-term effects of guided implantology on patient quality of life represents an important gap in the literature.

## 5. Conclusions

Guided oral implantology represents a valid, reliable, and precise alternative for the rehabilitation of tooth loss, offering advantages in terms of patient experience and comfort. Pain reduction is the most extensively studied variable; however, further research is needed to determine its impact on procedure duration and overall patient satisfaction. Rigorous methodological designs and long-term follow-up are essential to establish stronger evidence regarding its influence on patient perception and psychological well-being.

## Figures and Tables

**Table 1:** Table of search strategies.

Database	Search Strategy	Number of Articles
Medline	((“Dental Implants” OR “guided implant surgery”) AND (“Psychological Phenomena” OR “psychological impact” OR “mental health” OR “anxiety” OR “Quality of Life” OR “Stress”) AND (“Patient Satisfaction” OR “treatment outcomes”))	398
Cinhal	(MH “Dental Implants” OR “guided implant surgery”) AND (MH “Psychological Phenomena” OR “psychological impact” OR MH “Mental Health” OR MH “Anxiety” OR MH “Quality of Life” OR MH “Stress”) AND (MH “Patient Satisfaction” OR “treatment outcomes”)	103
Embase	(“dental implants”/exp OR “guided implant surgery”) AND (“psychological phenomena”/exp OR “psychological phenomena” OR “psychological impact”/exp OR “psychological impact”) AND (“patient satisfaction”/exp OR “patient satisfaction” OR “treatment outcomes”)	28

**Table 2:** Summary table of the characteristics of the included studies.

Author and Year of Publication (Country of Execution)	SD	Duration of the Study	SS (NI)	AA [Sex]	Funding [Type of Funding]	Conflict of Interest [Quality Rating]
Sancho-Puchades et al., 2019 [26] (Switzerland)	RCT	NR	73 (NR)	NR [NR]	Yes [Public: University of Geneva and University of Zurich]	No [2 Low]
Vercruyssen et al., 2014 [23] (Belgium)	RCT	1 year	59 (314)	Avg. 58 years [Both]	Yes [Private: Nobel Biocare]	No [3 Low]
Vercruyssen et al., 2014 [22] (Belgium)	RCT	7 days	59 (314)	Avg. 58 years [Both]	Yes [Private: Nobel Biocare]	No [2 Low]
Nirula et al., 2023 [28] (India)	RCT	4 months	60 (210)	Avg. 48 years [Both]	NR [NR]	No [2 Low]
Frizzera et al., 2011 [27] (Brazil)	RCT	3 months	10 (20)	Over 18 years old	Yes [Private: FAESA and FAPES and Derig-Implants]	No [5 High]
Montero et al., 2019 [18] (Spain)	N-RCT	6 months	104 (399)	Avg. 55 years [Both]	Yes [Public: University of Salamanca]	No [1 Low]
Merli et al., 2008 [19] (Italy)	CS	8 months	13 (89)	Avg. 62 years [Both]	Yes [Private: Nobel Biocare]	NR [66% Low]
Fortin et al., 2006 [20] (France)	CS	7 months	60 (152)	19–82 years [Both]	NR [NR]	NR [89% High]
Pozzi et al., 2014 [25] (Italy]	RCT	1 year	51 (202)	62.7–63.4 years [Both]	NR [NR]	No [3 Low]
Engkawong et al., 2021 [29] (Thailand)	RCT	2 weeks	88 (179)	32–74 years [Both]	NR [NR]	No [3 Low]
Lindeboom & Van Wijk, 2009 [21] (Netherlands)	CS	10 months	16 (96)	54–58 years [NR]	NR [NR]	NR [77.7% High]
Jorba-García et al., 2023 [24] (Spain)	RCT	8 months	29 (44)	59–61 years [Both]	NR [NR]	No. [3 Low]
Total			SS = 622 (NI) = (192)			

RCT: Randomized Clinical Trial; N-RCT: Non-randomized clinical trial; CS: Case series; SD: Study Design; SS: Sample Size; NI: Number of Implants Placed; AA: Average Age of Participants; NR: No reported; FAESA: Faculdades Integradas Espírito-Santenses; FAPES: Fundação de Amparo à Pesquisa e Inovação no Espírito Santo.

**Table 3:** Table of results of the clinical variables of the included studies.

Author	Comparison Group	[Intervention] Flap	Features/Software	Load
Sancho-Puchades et al. [26]	Conventional and static guided surgery with radiological splint and surgical splint and guided surgery with surgical splint	[Static] Flap: all groups	Simplant Dentsply Sirona software. Bone regeneration and bone elevation, if necessary	NR
Vercruyssen et al. [23]	conventional and static guided surgery	[Static] Without flap: mucosal support groups (Mat Mu and Fac Mu). With flap: Materialize Universal/bone (Mat Bo), Facilitate/bone (Fac Bo), freehand navigation (Freehand), and pilot drill template (Templ)	It uses two guided surgery systems: Materialize Universal (Materialize, Leuven, Belgium) and FacilitateTM (DENTSPLY Implants, Molndal, Sweden) with depth control stops	3–4 months
Vercruyssen et al. [22]	Conventional and static guided surgery	[Static] Without flap: mucosal support groups (Mat Mu and Fac Mu). With flap: Materialize Universal/bone (Mat Bo), Facilitate/bone (Fac Bo), freehand navigation (Freehand), and pilot drill template (Templ)	It uses two guided surgery systems: Materialize Universal (Materialize, Leuven, Belgium) and FacilitateTM (DENTSPLY Implants, Molndal, Sweden) with depth control stops	3–4 months
Nirula, P. et al. [28]	Conventional and dynamic guided surgery	[Dynamic] Flap: conventional surgery	Navident Software (Toronto, ON, Canada)	NR
Frizzera et al. [27]	Conventional and static guided surgery.	[Static] Flap: conventional surgery	Blue sky plan Software (Libertyville, IL, USA)	NR
Montro et al. [18].	Conventional, static guided surgery with conventional loading and immediate loading surgery	[Static] Flap: conventional surgery	MozoGrau Guided Surgery Software (MG_Fidelis, Mozograu, Valladolid, Spain)	Immediate and conventional
Merli et al. [19]	Static guided surgery.	[Static] Flap: 2 patients for bone regeneration	Nobel Biocare, Goteborg, Sweden	Immediate and conventional
Fortin et al. [20]	Conventional and static guided surgery.	[Static] Flap: conventional surgery	CADimplant v2.3 Software	NR
Pozzi et al. [25]	Conventional and static guided surgery.	[Static] Flapless or mini flap	Software Nobel Biocare, Kloten, Switzerland	Immediate.
Engkawong et al. [29]	Conventional, static guided surgery and dynamic guided surgery.	[Static and dynamic] With and without flap depending on the consistency of keratinized mucosa	coDiagnostiX software, Dental Wings, Inc., Montreal, QC, Canada, and Iris-100, EPED, Inc., Taiwan. Bone regeneration and bone elevation, if necessary	NR
Lindeboom & Van Wijk [21]	Flap-guided surgery and flapless-guided surgery.	[Static] Flap: guided surgery; Flapless: guided surgery	Nobel Biocare AB Software, Goteborg, Sweden	NR
Jorba-García et al. [24]	Conventional and dynamic guided surgery.	[Dynamic] Flap-free whenever possible for both groups	Navident Software (Navident^®^, ClaroNav Technology Inc.^®^; Los Ángeles, CA, USA)	NR

NR: not reported.

**Table 4:** Results table of the instruments used to evaluate patient perception.

Study	OHIP	VAS-p	VAS-ql	HRQoL	MPQ-DLV	Likert-a	PREM’s	IES-R	s-DAI	O-3	Self-d
Sancho-Puchades et al. [26]		X	X								
Vercruyssen et al. [23]	X										
Vercruyssen et al. [22]		X		X	X						
Nirula et al. [28]		X					X				
Frizzera et al. [27]		X	X								
Montero et al. [18]			X	X							
Merli et al. [19]											X
Fortin et al. [20]		X									
Pozzi et al. [25]		X								X	X
Engkawong et al. [29]		X				X					
Lindeboom & Van Wijk [21]	X							X	X		
Jorba-García et al. [24]	X	X									

OHIP: Oral Health Impact Profile; VAS-p: Visual Analog Scale (pain); VAS-ql: Visual Analog Scale (quality of life); HRQoL: Health-related quality of life instrument; MPQ-DLV: McGill Pain Questionnaire; Likert-a: Likert scale adapted; PREM’s: Patient Related Experience measures; IES-R: Impact of Event Scale-Revised; s-DAI: Dental Anxiety; O-3: Ordinary Pain Scale; Self-d: Self-developed questionnaire; X: It has been done.

**Table 5:** Results table for the validation of treatment perception.

Author	Instrument	Aspects Assessed	Questionnaire Variables	Time of Questionnaire Administration
Sancho-Puchades et al. [26]	Visual Analogue Scale (VAS) (pain). Visual Analogue Scale (VAS) (quality of life)	Intraoperative comfort, perceived duration of the surgical procedure, and intra- and postoperative symptoms	Swelling, bruising, bleeding, nausea, opening, chewing, social interaction, sleep, etc.	Immediately after surgery
Vercruyssen et al. [23]	Oral Health Impact Profile (OHIP 49)	Quality of life and patient satisfaction	Domains: Functional limitation, physical pain, psychological discomfort, physical disability, social disability and other reasons for discomfort	Preoperative and one year after receiving it
Vercruyssen et al. [22]	McGill Pain Questionnaire (MPQ-DLV). Oral Health-related Quality of Life (OHQoL). Visual Analogue Scale (VAS) (pain)	Postoperative pain and quality of life	Pain description and scale. Quality of life variables (masticatory function)	During the following 7 days postoperatively
Nirula et al. [28]	Patient-Related Experience measures (PREMs). Visual Analogue Scale (VAS) (pain)	Expectations, treatment satisfaction and postoperative pain	Experience with robotics, pain perception, and comfort during surgery	Preoperative and during the postoperative period
Frizzera F et al. [27]	Visual Analogue Scale (VAS) (pain) Visual Analogue Scale (VAS) (satisfaction)	Treatment satisfaction and pain	Intraoperative pain, postoperative pain, treatment satisfaction and surgical time	Preoperative and during the following 7 days postoperatively
Montero et al. [18]	Oral Health-related Quality of Life (OHQoL). Visual Analogue Scale (VAS) (satisfaction)	Quality of life and patient satisfaction	Impact on basic daily activities: eating, speaking, oral hygiene, sleeping, smiling, job functions, emotional stability, and social contact	Preoperative and at 6 months after surgery
Merli et al. [19]	Self-developed questionnaire	Patient satisfaction	Quality of life and cost	7 days after the provisional treatment and 30 days after definitive rehabilitation
Fortin et al. [20]	Visual Analogue Scale (VAS) (pain)	Postoperative pain	Postoperative pain and medication	During the following 7 days postoperatively
Pozzi et al. [25]	Standard pain scale (0–3). Self-reported questionnaires (satisfaction)	Postoperative pain and patient satisfaction	Function, aesthetics, and comfort	Pain: 3 days after treatment
Engkawong et al. [29]	Visual Analogue Scale (VAS) (pain) 5-point Likert scale adapted de Yao and colleagues (satisfaction)	Postoperative pain and patient satisfaction	Pain, bleeding, difficulty chewing and speaking, oral hygiene, surgical time	1–2 weeks after treatment
Lindeboom & Van Wijk [21]	Impact of Event Scale-Revised (IES-R) Dental anxiety (s-DAI) Oral Health Impact Profile 14 (OHIP-14)	Patient satisfaction, quality of life and dental anxiety	Pain, anxiety, duration of treatment	During the following 7 days postoperatively
Jorba-García et al. [24]	Oral Health Impact Profile 14 (OHIP-14) Visual Analogue Scale (VAS)	Quality of life, patient satisfaction and pain	Patient perception, treatment recommendation, intra- and postoperative pain, surgical time	Preoperatively, at the end of surgery, and 7 days postoperatively

**Table 6:** Results table of the conclusions of the studies.

Study	Conclusions
Sancho-Puchades et al. [26]	Overall, patients preferred computer-based technologies. No differences were observed in intra- or postoperative discomfort compared to control protocols. More extensive surgical procedures negatively affected intra- and postoperative quality of life, regardless of treatment group.
Vercruyssen et al. [23]	No differences were found at 1-year follow-up between implant and patient outcome variables for guided vs. conventional treatments. Guided surgery appears to be a valid and predictable treatment option.
Vercruyssen et al. [22]	Few differences were found in patient outcome variables across treatment groups. However, patients undergoing conventional flap implant placement tended to experience pain for a longer period.
Nirula et al. [28]	Immediate postoperative pain perception and comfort levels were better with dynamic navigation. Patients showed strong interest in robotic dentistry, appreciating the ability to visualize and understand the procedure on screen.
Frizzera et al. [27]	Flapless guided implant surgeries performed by inexperienced clinicians showed reduced surgical time and yielded better patient-reported outcomes both intra- and postoperatively, along with reduced medication usage compared to conventional implant techniques.
Montero et al. [18]	Clear improvement in oral well-being was observed after implant therapy. OHQoL scores and patient satisfaction were notably higher in patients treated with guided surgery and immediate loading protocols, although all groups ultimately achieved similar well-being levels.
Merli et al. [19]	Guided implant surgery software can be very helpful in planning and managing complex cases. However, it requires progressive training. The entire flapless and immediate loading process is not simple and should only be used by experienced operators.
Fortin et al. [20]	With flapless procedures, patients experienced less intense and shorter-duration pain.
Pozzi et al. [25]	When treatment planning was perfomed with CBCT scanning using dedicated 3D planning software, no statistically significant differences were observed between computer-guided and freehand rehabilitations—except that freehand sites, where flaps were more often raised, showed more postoperative pain and inflammation.
Engkawong et al. [29]	Conventional freehand, static, and dynamic CAIS techniques for dental implant surgery did not result in any difference in postoperative pain and inflammation levels and appeared to lead to equal levels of patient satisfaction.
Lindeboom & Van Wijk [21]	The flap procedure group reported less impact on quality of life and included more patients who reported no pain at all during placement.
Jorba-García et al. [24]	d-CAIS systems significantly improve implant placement accuracy in partially edentulous patients compared to the manual approach. However, they significantly increase surgical time and do not appear to enhance patient satisfaction or reduce postoperative pain.

## Data Availability

The original contributions presented in this study are included in the article. Further inquiries can be directed to the corresponding authors.

## References

[1] Marshall S., Haywood K., Fitzpatrick R. (2006). Impact of patient-reported outcome measures on routine practice: A structured review. J. Eval. Clin. Pract..

[2] Allen P.F. (2003). Assessment of oral health related quality of life. Health Qual. Life Outcomes.

[3] McGrath C., Bedi R. (2004). A national study of the importance of oral health to life quality to inform scales of oral health related quality of life. Qual. Life Res..

[4] Stucki G. (2005). International Classification of Functioning, Disability, and Health (ICF): A Promising Framework and Classification for Rehabilitation Medicine. Am. J. Phys. Med. Rehabil..

[5] Knezović Zlatarić D., Čelebić A., Valentić-Peruzović M. (2002). The effect of removable partial dentures on periodontal health of abutment and non-abutment teeth. J. Periodontol..

[6] Cunha L.D.A.P., Pellizzer E.P., Verri F.R., Pereira J.A. (2008). Evaluation of the influence of location of osseointegrated implants associated with mandibular removable partial dentures. Implant Dent..

[7] Velasco-Ortega E., Jiménez-Martin I.R., Moreno-Muñoz J., Nuñez-Márquez E., Rondón-Romero J.L., Cabanillas-Balsera D., Jiménez-Guerra A., Ortiz-Garcia I., López-López J., Monsalve-Guil L. (2022). Long-term treatment outcomes of implant prostheses in partially and totally edentulous patients. Materials.

[8] AL-Omiri M., Hantash R.A., AL-Wahadni A. (2005). Satisfaction with dental implants: A literature review. Implant Dent..

[9] Monsalve-Guil L., Velasco-Ortega E., Ortiz-Garcia I., Matos-Garrido N., Moreno-Muñoz J., Núñez-Márquez E., Rondón-Romero J.L., López-López J., Jiménez-Guerra Á. (2025). Retrospective clinical follow-up of implants placed in edentulous jaws after computer-guided surgery and immediate loading, in geriatric patients. Med. Oral Patol. Oral Cir. Bucal.

[10] Rouzé l’Alzit F., Cade R., Naveau A., Babilotte J., Meglioli M., Catros S. (2022). Accuracy of commercial 3D printers for the fabrication of surgical guides in dental implantology. J. Dent..

[11] Tahmaseb A., Wismeijer D., Coucke W., Derksen W. (2014). Computer technology applications in surgical implant dentistry: A systematic review. Int. J. Oral Maxillofac. Implant..

[12] Zhao W., Teng W., Su Y., Zhou L. (2024). Accuracy of dental implant surgery with freehand, static computer-aided, dynamic computer-aided, and robotic computer-aided implant systems: An in vitro study. J. Prosthet. Dent..

[13] Fonteyne E., De Bruyn H., De Fruyt F. (2020). Quality of life and social participation in dental rehabilitation: A personality and multi-informant perspective. J. Dent..

[14] Page M.J., McKenzie J.E., Bossuyt P.M., Boutron I., Hoffmann T.C., Mulrow C.D., Shamseer L., Tetzlaff J.M., Akl E.A., Brennan S.E. (2021). The PRISMA 2020 statement: An updated guideline for reporting systematic reviews. BMJ.

[15] von Elm E., Altman D.G., Egger M., Pocock S.J., Gøtzsche P.C., Vandenbroucke J.P. (2014). The STROBE Statement: Guidelines for reporting observational studies. Int. J. Surg..

[16] Clark H.D., Wells G.A., Huët C., McAlister F.A., Salmi L.R., Fergusson D., Laupacis A. (1999). Assessing the quality of randomized trials: Reliability of the Jadad scale. Control. Clin. Trials.

[17] Munn Z., Barker T.H., Moola S., Tufanaru C., Stern C., McArthur A., Stephenson M., Aromataris E. (2020). Methodological quality of case series studies: An introduction to the JBI critical appraisal tool. JBI Evid. Synth..

[18] Montero J., Dolz J., Silvestre F.J., Flores J., Dib A., Gómez-Polo C. (2019). Changes in oral health-related quality of life after three different strategies of implant therapy: A clinical trial. Odontology.

[19] Merli M., Bernardelli F., Esposito M. (2008). Computer-guided flapless placement of immediately loaded dental implants in the edentulous maxilla: A pilot prospective case series. Eur. J. Oral Implantol..

[20] Fortin T., Bosson J.L., Isidori M., Blanchet E. (2006). Effect of flapless surgery on pain experienced in implant placement using an image-guided system. Int. J. Oral Maxillofac. Implant..

[21] Lindeboom J.A., Van Wijk A.J. (2010). A comparison of two implant techniques on patient-based outcome measures: A report of flapless vs. conventional flapped implant placement. Clin. Oral Implant. Res..

[22] Vercruyssen M., De Laat A., Coucke W., Quirynen M. (2014). An RCT comparing patient-centred outcome variables of guided surgery (bone- or mucosa-supported) with conventional implant placement. J. Clin. Periodontol..

[23] Vercruyssen M., Van De Wiele G., Teughels W., Naert I., Jacobs R., Quirynen M. (2014). Implant- and patient-centred outcomes of guided surgery, a 1-year follow-up: An RCT comparing guided surgery with conventional implant placement. J. Clin. Periodontol..

[24] Jorba-García A., Bara-Casaus J.J., Camps-Font O., Sánchez-Garcés M.Á., Figueiredo R., Valmaseda-Castellón E. (2023). Accuracy of dental implant placement with or without the use of a dynamic navigation-assisted system: A randomized clinical trial. Clin. Oral Implant. Res..

[25] Pozzi A., Tallarico M., Moy P.K. (2016). Four-implant overdenture fully supported by a CAD-CAM titanium bar: A single-cohort prospective 1-year preliminary study. J. Prosthet. Dent..

[26] Sancho-Puchades M., Alfaro F., Naenni N., Jung R., Hämmerle C., Schneider D. (2019). A randomized controlled clinical trial comparing conventional and computer-assisted implant planning and placement in partially edentulous patients. Part 2: Patient-related outcome measures. Int. J. Periodontics Restor. Dent..

[27] Frizzera F., Calazans N.N.N., Pascoal C.H., Martins M.E., Mendonça G. (2021). Flapless guided implant surgeries compared with conventional surgeries performed by nonexperienced individuals: Randomized and controlled split-mouth clinical trial. Int. J. Oral Maxillofac. Implant..

[28] Nirula P., Selvaganesh S., Thiyaneswaran N. (2023). Feedback on dental implants with dynamic navigation versus freehand. Bioinformation.

[29] Engkawong S., Mattheos N., Pisarnturakit P.P., Pimkhaokham A., Subbalekha K. (2021). Comparing patient-reported outcomes and experiences among static, dynamic computer-aided, and conventional freehand dental implant placement: A randomized clinical trial. Clin. Implant. Dent. Relat. Res..

[30] Campos L.A., Peltomäki T., Marôco J., Campos J.A.D.B. (2021). Use of Oral Health Impact Profile-14 (OHIP-14) in different contexts: What is being measured?. Int. J. Environ. Res. Public Health.

[31] Sischo L., Broder H.L. (2011). Oral health-related quality of life: What, why, how, and future implications. J. Dent. Res..

[32] Delgado D.A., Lambert B.S., Boutris N., McCulloch P.C., Robbins A.B., Moreno M.R., Harris J.D. (2018). Validation of digital visual analog scale pain scoring with a traditional paper-based visual analog scale in adults. J. Am. Acad. Orthop. Surg. Glob. Res. Rev..

[33] Black N., Varaganum M., Hutchings A. (2014). Relationship between patient-reported experience (PREMs) and patient-reported outcomes (PROMs) in elective surgery. BMJ Qual. Saf..

[34] Seymour R.A., Charlton J.E., Phillips M.E. (1983). An evaluation of dental pain using visual analogue scales and the Mcgill Pain Questionnaire. J. Oral Maxillofac. Surg..

[35] Horowitz M., Wilner N., Alvarez W. (1979). Impact of Event Scale: A measure of subjective stress. Psychosom. Med..

[36] Kvale G., Berg E., Raadal M. (1998). The ability of Corah’s Dental Anxiety Scale and Spielberger’s State Anxiety Inventory to distinguish between fearful and regular Norwegian dental patients. Acta Odontol. Scand..

[37] Yao C.J., Cao C., Bornstein M.M., Mattheos N. (2018). Patient-reported outcome measures of edentulous patients restored with implant-supported removable and fixed prostheses: A systematic review. Clin. Oral Implant. Res..

[38] Yeo X.H., Uei L.J., Yi M., Kungsadalpipob K., Subbalehka K., Al-Nawas B., Mattheos N. (2025). Computer-Assisted Implant Surgery: Patients’ Experience and Perspectives. Clin. Exp. Dent. Res..

[39] Lanis A., Peña-Cardelles J.F., Negreiros W.M., Hamilton A., Gallucci G.O. (2024). Impact of digital technologies on implant surgery in fully edentulous patients: A scoping review. Clin. Oral Implant. Res..

[40] Ashy L.M. (2021). Clinicians’ Attitude Toward Computer-Guided Implant Surgery Approach: Survey in Saudi Arabia. Pragmat. Obs. Res..

[41] Alnafaiy S.M., Alyousef H., Aljabr R., Tounsi A., Almutairi R., Albaijan R.S. (2024). Digital technology implementation in prosthodontics postgraduate programs in Saudi Arabia: A multi-institutional survey of program directors. BMC Oral Health.

[42] Pjetursson B.E., Tan W.C., Zwahlen M., Lang N.P. (2008). A systematic review of the success of sinus floor elevation and survival of implants inserted in combination with sinus floor elevation: Part I: Lateral approach. J. Clin. Periodontol..

[43] Pommer B., Zechner W., Watzak G., Ulm C., Watzek G., Tepper G. (2011). Progress and trends in patients’ mindset on dental implants. II: Implant acceptance, patient-perceived costs and patient satisfaction. Clin. Oral Implant. Res..

[44] Joda T., Brägger U. (2015). Digital vs. conventional implant prosthetic workflows: A cost/time analysis. Clin. Oral Implant. Res..

[45] Aghaloo T.L., Moy P.K. (2007). Which hard tissue augmentation techniques are the most successful in furnishing bony support for implant placement?. Int. J. Oral Maxillofac. Implant..

[46] Sankar H., Shalini M., Rajagopalan A., Gupta S., Kumar A., Shouket R. (2025). Dental implant placement accuracy with robotic surgery compared to free-hand, static and dynamic computer assisted techniques: Systematic review and meta-analysis. J. Oral Biol. Craniofac. Res..

[47] Schiavon L., Mancini L., Settecase E., Jung R.E., Joda T. (2025). Does Computer-Assisted Surgery Improve the Accuracy of Immediate Implant Placement? A Systematic Review and Network Meta-Analysis. J. Periodontal Res..

[48] Mittal H., John M.T., Sekulić S., Theis-Mahon N., Rener-Sitar K. (2019). Patient-Reported Outcome Measures for Adult Dental Patients: A Systematic Review. J. Evid. Based Dent. Pract..

[49] Hua F. (2023). Dental patient-reported outcomes update 2022. J. Evid. Based Dent. Pract..

[50] Schierz O., Reissmann D.R. (2021). Dental patient-reported outcomes—The promise of dental implants. J. Evid. Based Dent. Pract..

[51] Omara M., Stamm T., Bekes K. (2023). Lessons learned from the first steps of implementing value-based oral health care: A case study from the Medical University of Vienna. J. Evid. Based Dent. Pract..

[52] Reissmann D.R. (2021). Methodological considerations when measuring oral health-related quality of life. J. Oral Rehabil..

[53] Chanthavisouk P., John M.T., Paulson D., Pattanaik S. (2022). Commonalities among dental patient-reported outcomes (dPROs)-A Delphi consensus study. PLoS ONE.

[54] Ala L.A.B., Nogueira T.E., Leles C.R. (2022). One-year prospective study on single short (7-mm) implant overdentures in patients with severely resorbed mandibles. Clin. Oral Implant. Res..

[55] Thoma D.S., Strauss F.J., Mancini L., Gasser T.J.W., Jung R.E. (2023). Minimal invasiveness in soft tissue augmentation at dental implants: A systematic review and meta-analysis of patient-reported outcome measures. Periodontology 2000.

[56] Kunavisarut C., Santivitoonvong A., Chaikantha S., Pornprasertsuk-Damrongsri S., Joda T. (2022). Patient-reported outcome measures comparing static computer-aided implant surgery and conventional implant surgery for single-tooth replacement: A randomized controlled trial. Clin. Oral Implant. Res..

[57] Abou-Ayash S., Fonseca M., Pieralli S., Reissmann D.R. (2023). Treatment effect of implant-supported fixed complete dentures and implant overdentures on patient-reported outcomes: A systematic review and meta-analysis. Clin. Oral Implant. Res..

